# Inferior alveolar nerve repositioning and securing in conjunction with dental implant placement: a technical note

**DOI:** 10.1186/s40729-020-00268-w

**Published:** 2020-11-18

**Authors:** George Deryabin, Simonas Grybauskas

**Affiliations:** 1 Chicago, USA; 2 S’OS Orthognathic Surgery, Vytenio 22-201, Vilnius, Lithuania

**Keywords:** Inferior alveolar nerve transposition, Vestibuloplasty, Dental implants, Titanium screws

Loss of teeth in the posterior mandible leads to progressive alveolar bone resorption, diminishing the amount of attached and unattached mucosa and superficial location of the inferior alveolar nerve (IAN) [1]. Applying dental implants in cases with these bone defects may require multiple surgeries, including vestibuloplasty and soft tissue grafting during the final treatment stages [2]. IAN transposition or lateralisation in combination with dental implant placement is a seldom-used treatment modality due to its complexity and potential IAN damage. However, these techniques could be successfully utilised in cases where there is less than 10 mm bone height above the canal and the quality of cancellous bone is poor [3]. There are insufficient data on how the IAN should be handled and secured to minimise its long-term morbidity [4]. In certain IAN repositioning cases, it is left covered with only a thin layer of bone or soft tissues. Therefore, vestibuloplasty at the time of implant uncovering could pose a real challenge to salvaging the IAN during mucosal separation.

The purpose of this article was to demonstrate a technique utilising titanium screws as IAN markers during its repositioning. These screws serve as landmarks that allow a clinician to easily locate the IAN during vestibuloplasty or placement of healing abutments.

The operation is performed under a combination of local anaesthesia with IV sedation. A crestal incision is made in the keratinised gingiva of the edentulous area. It is initiated in the retromolar region at the mucogingival junction and carried out toward the distal aspect of the most posterior tooth. A full-thickness flap is elevated, exposing the alveolar crest and lateral body of the mandible to the mental foramen. A full corticotomy is performed with a piezoelectric saw creating a rectangular bony window lateral to the inferior alveolar canal according to the IAN pathway that was determined by preoperative computed tomography scan (Figs. 1, 2, 3). The bony window is removed, exposing the neurovascular bundle within the inferior alveolar canal. The neurovascular bundle is mobilised and retracted laterally while implants are installed (Fig. 4). In cases of nerve transposition, the osteotomy window is extended anteriorly to expose the mental foramen and incisive branch. Then, the incisive branch is transected, and the IAN is freed from the canal and displaced laterally.

**Fig. 1:**
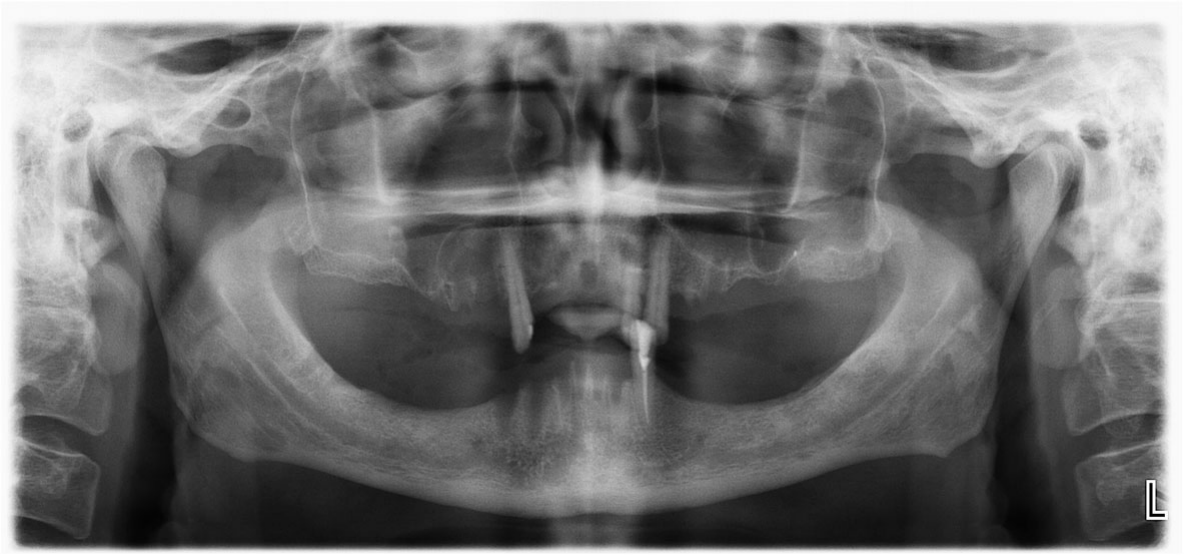
A panoramic radiography before surgery

**Fig. 2:**
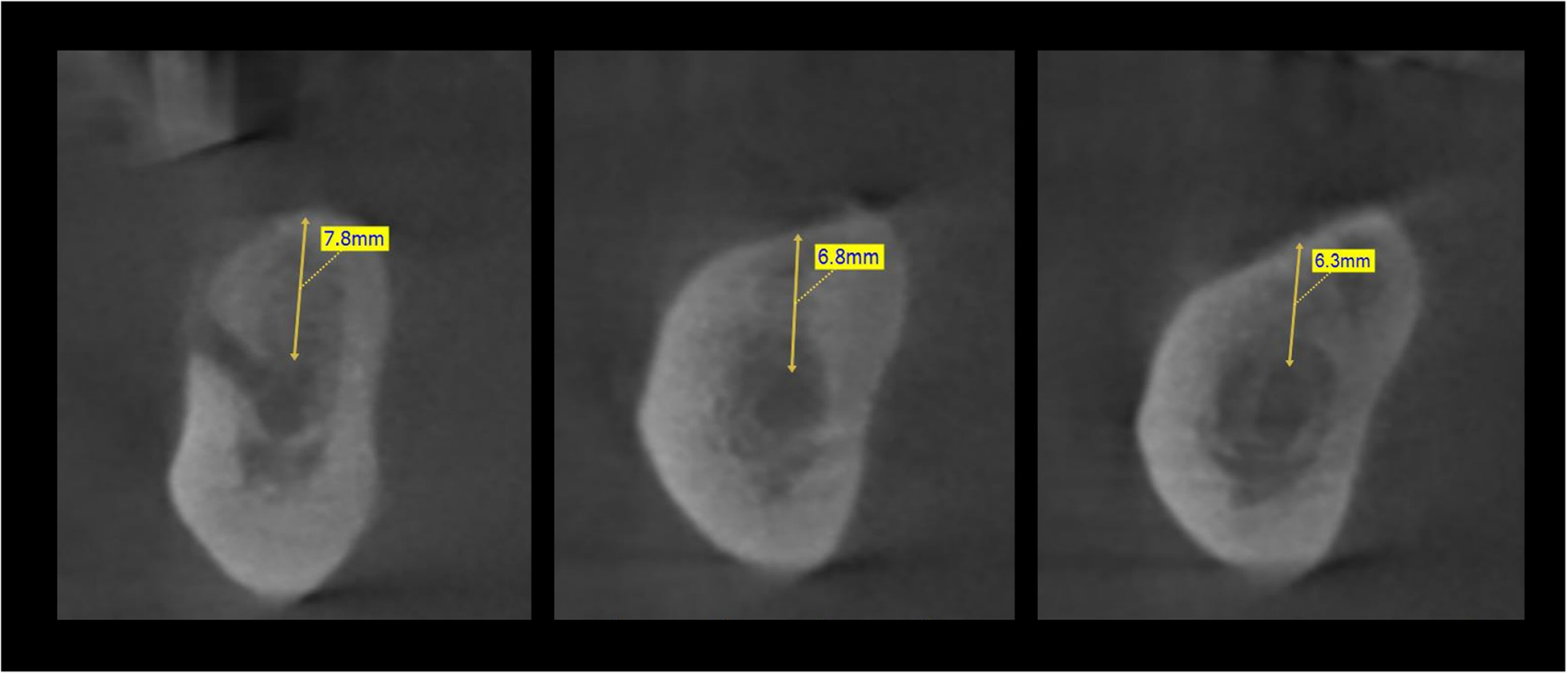
A CT scan before surgery

**Fig. 3:**
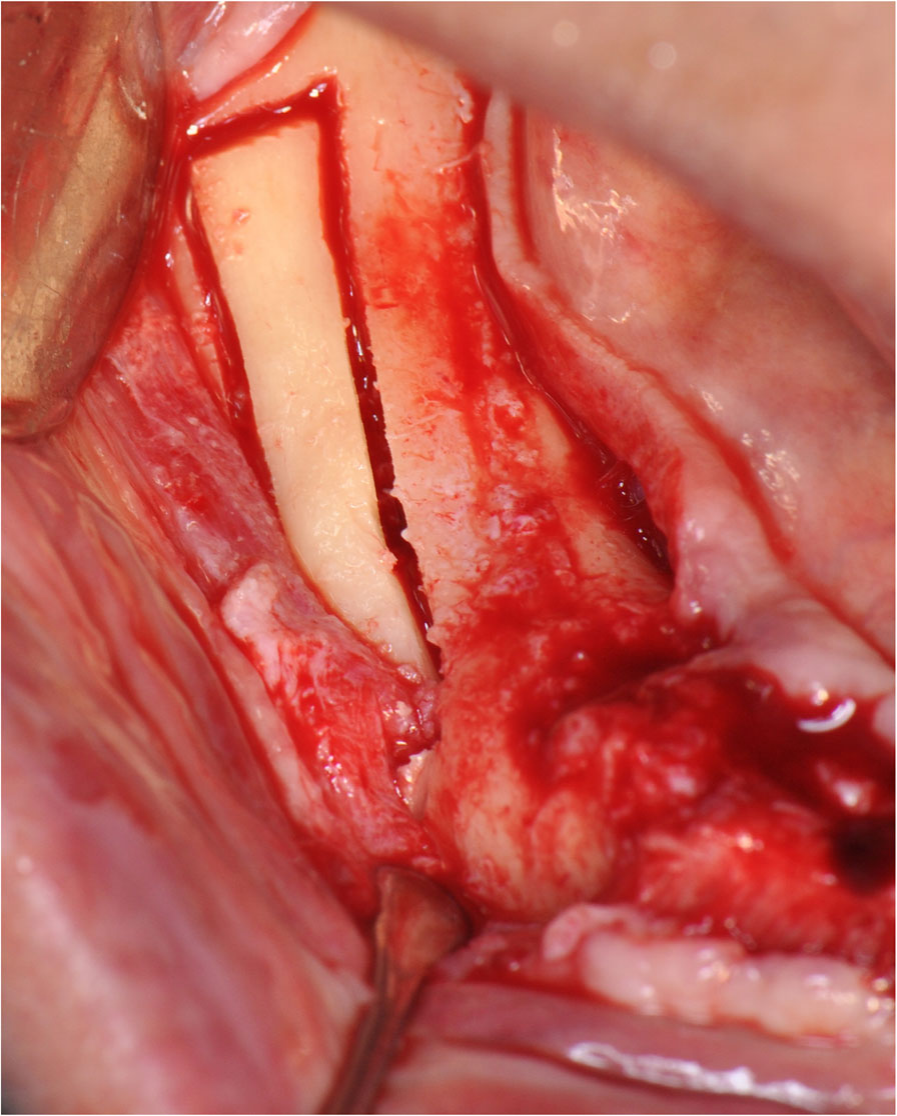
A full corticotomy is performed creating a rectangular bony window lateral to the inferior alveolar canal

**Fig. 4:**
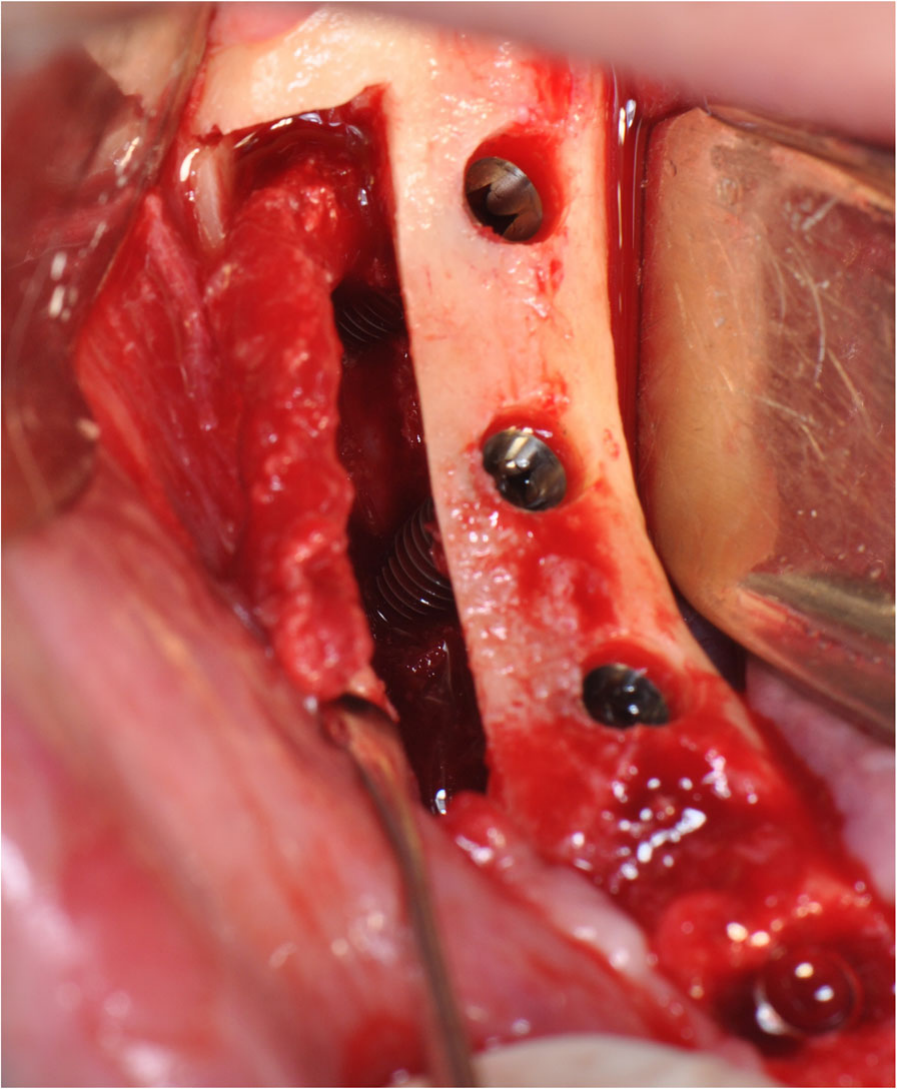
The neurovascular bundle is mobilised and retracted laterally while the implants are installed

A titanium mini-screw is then placed halfway in the lateral side of the mandible at the upper osteotomy line (Fig. 5). The screws offer two benefits: they serve as a passive barrier to the IAN and prevent it from migrating and the screws indicate the IAN’s new location in radiological studies for identification during re-entry surgery (Figs. 6, 7). The titanium screws provide a convenient landmark for a clinician, minimising risks of IAN damage during the placement of healing abutments and/or vestibuloplasty (Figs. 8, 9, 10, 11, 12).

**Fig. 5:**
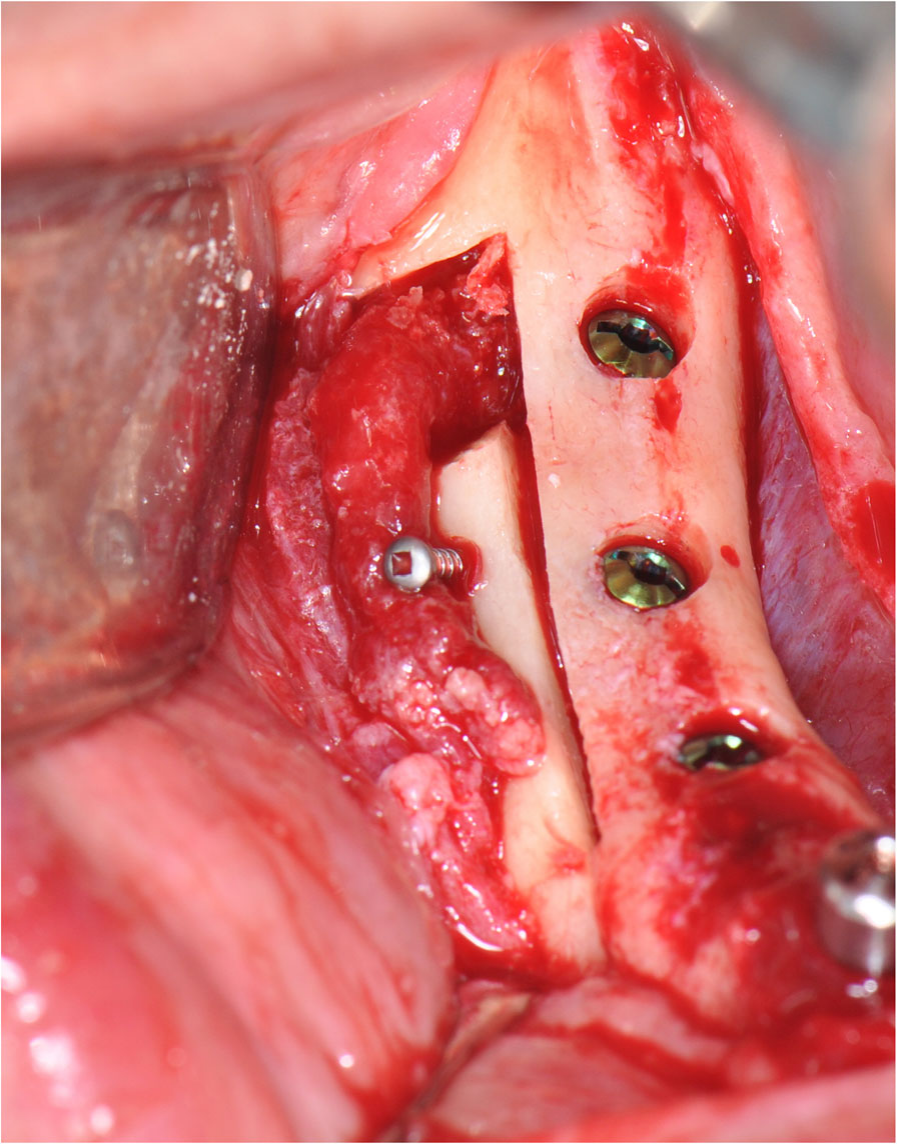
A titanium mini-screw is placed halfway on the lateral side of the mandible at the upper osteotomy line

**Fig. 6:**
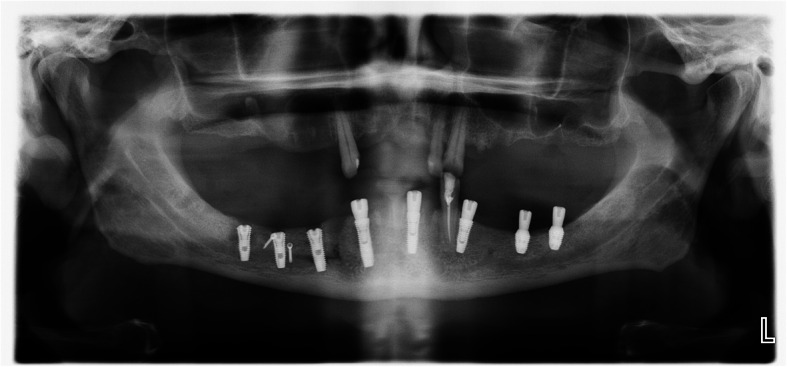
A panoramic radiography after IAN transposition and implant placement

**Fig. 7:**
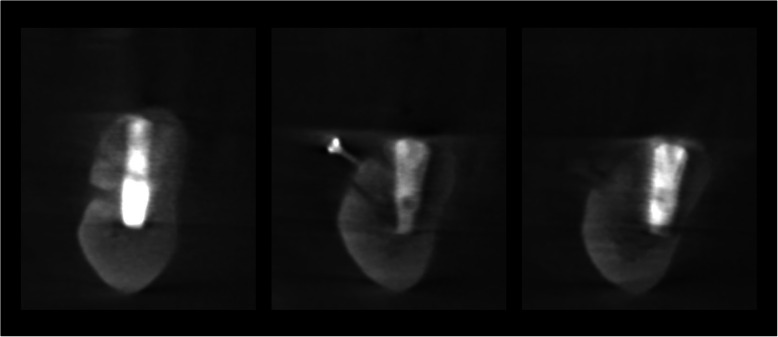
A CT scan after surgery

**Fig. 8:**
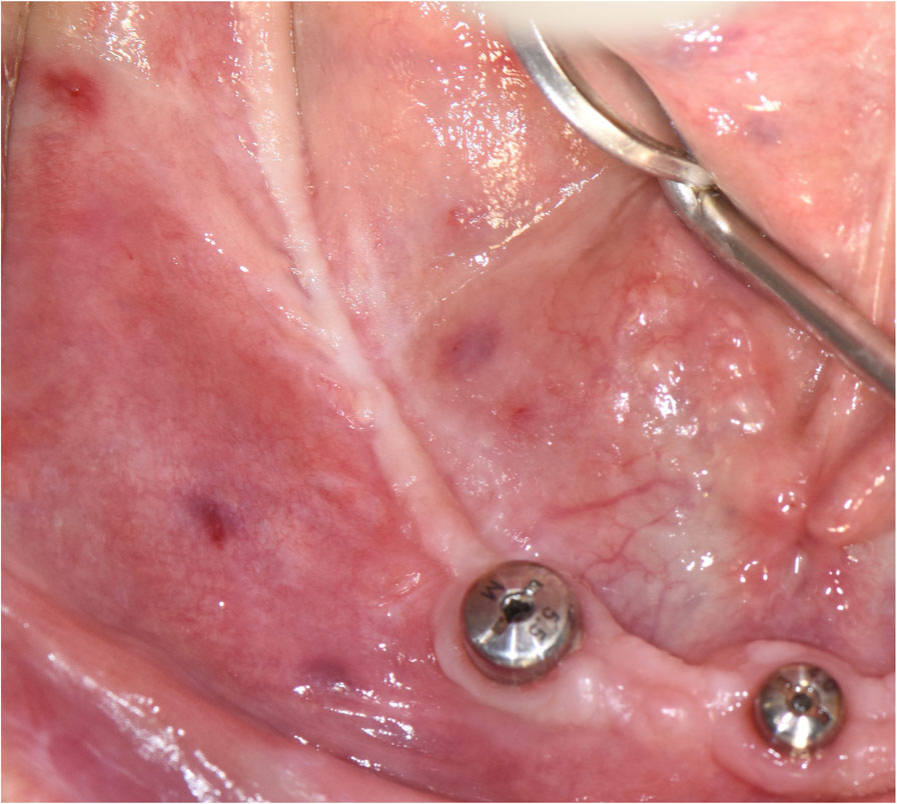
Narrow band of keratinised gingiva at the second-stage surgery

**Fig. 9:**
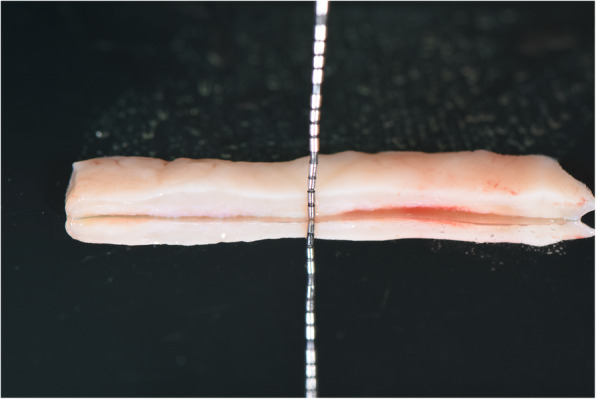
A free gingival graft is obtained from the palate

**Fig. 10:**
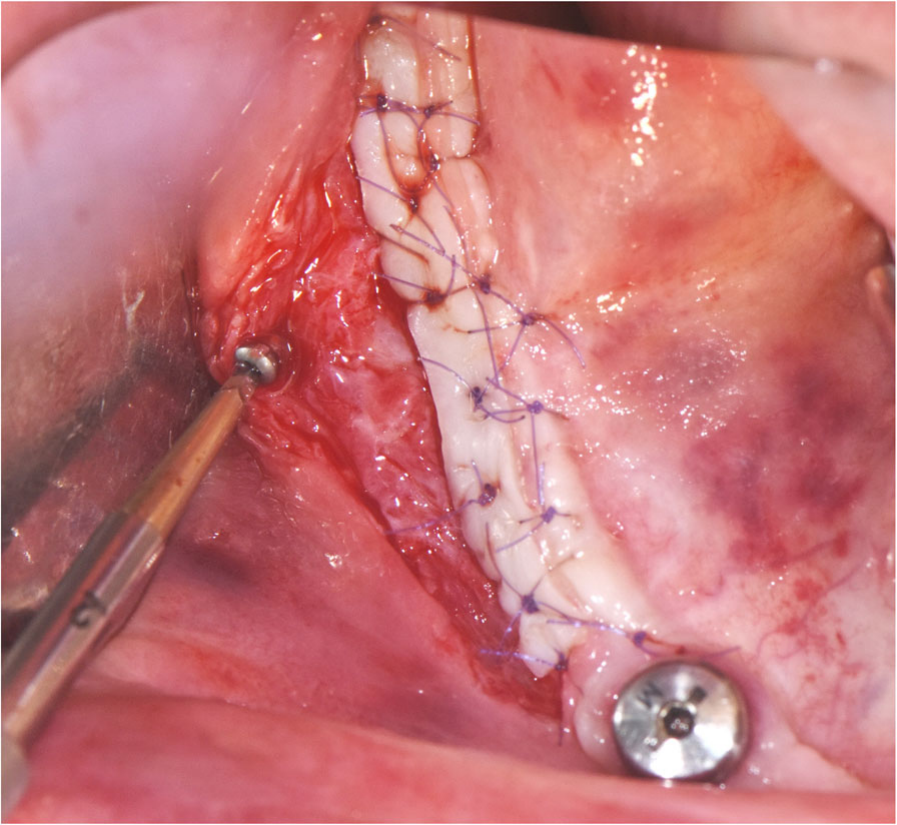
Vestibuloplasty with free gingival graft is performed. Notice the titanium mini-screw that indicates the neurovascular bundle’s position

**Fig. 11:**
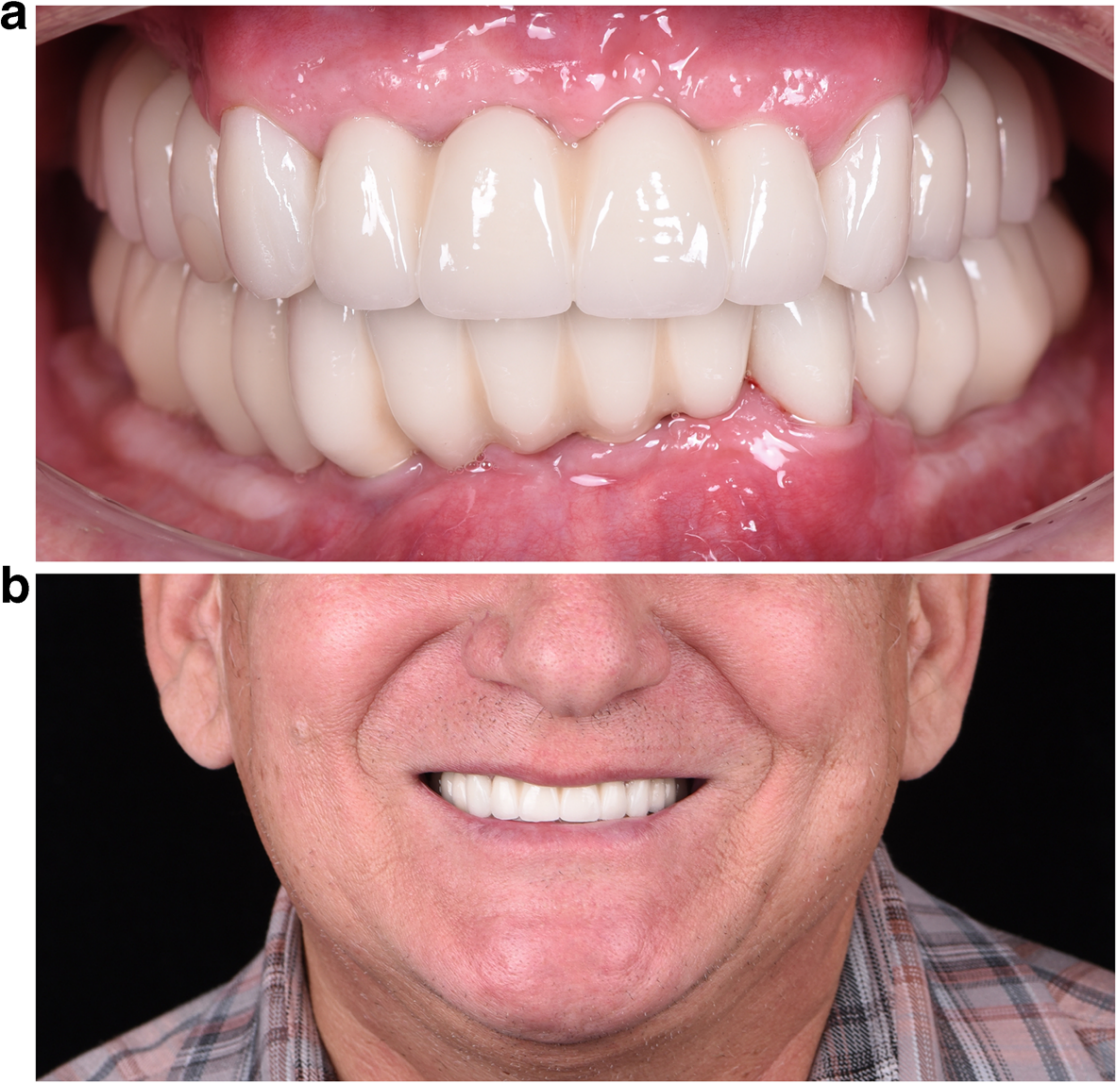
**a**, **b** Final result (Prosthodontist: Evgeniy Shor)

**Fig. 12:**
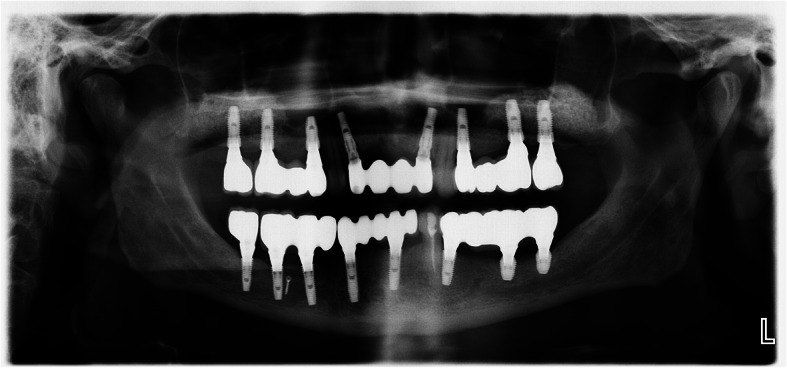
A panoramic radiography after the delivery of the final prosthesis

The space between the implants and repositioned neurovascular bundle is grafted, and the mucoperiosteal flaps are repositioned and sutured with resorbable sutures. Six months of healing time is allowed before second-stage surgery.

This technique was successfully implemented in 15 consecutive cases.

## Data Availability

Not applicable

## Abbreviations

IAN: Inferior alveolar nerve
IV sedation: Intravenous sedation
